# Circulating CD14^+^CD163^+^CD206^+^ M2 Monocytes Are Increased in Patients with Early Stage of Idiopathic Membranous Nephropathy

**DOI:** 10.1155/2018/5270657

**Published:** 2018-06-21

**Authors:** Jie Hou, Manli Zhang, Yuhong Ding, Xinrui Wang, Tao Li, Pujun Gao, Yanfang Jiang

**Affiliations:** ^1^Department of Nephrology, The First Hospital of Jilin University, Changchun 130021, China; ^2^Department of Hepatology and Gastroenterology, The Second Part of First Hospital of Jilin University, Changchun 130021, China; ^3^Genetic Diagnosis Center, The First Hospital of Jilin University, Changchun 130021, China; ^4^Department of Hepatology, The First Hospital of Jilin University, Changchun 130021, China; ^5^Key Laboratory of Zoonosis Research, Ministry of Education, The First Hospital of Jilin University, Changchun 130021, China; ^6^Jiangsu Co-Innovation Center for Prevention and Control of Important Animal Infectious Diseases and Zoonoses, Yangzhou 225009, China

## Abstract

**Aim:**

To analyze changes in peripheral blood monocytes and their clinical significance in patients with early stage of idiopathic membranous nephropathy (IMN).

**Methods:**

A total of 27 patients with early stage of IMN and 16 age- and sex-matched healthy controls (HCs) were recruited for the study. The monocyte subset counts in circulation were measured by flow cytometry, and serum interleukin- (IL-) 10 and IL-12 concentrations were tested by enzyme-linked immunosorbent assay. The potential association between clinical signs and monocyte subset counts was analyzed statistically.

**Results:**

Compared with the HCs, the patients with early stage of IMN had higher counts of CD14^+^CD163^+^, CD14^+^CD163^+^CD206^+^, and CD14^+^CD163^+^CD206^+^CD115^+^ M2-like monocytes. The CD14^+^CD163^+^CD206^+^ M2-like cell counts and intracellular IL-10 concentrations in the monocytes were positively correlated with progression in proteinuria. The levels of serum IL-10 were significantly higher in early IMN patients than in the HCs. Furthermore, CD14^+^CD163^+^CD206^+^ M2-like cell counts in the patients with incipient IMN were also positively related with 24 h urinary albumin levels and the values of serum M-type phospholipase A2 receptor (PLA2R).

**Conclusion:**

CD14^+^CD163^+^CD206^+^ M2-like monocytes may contribute to the pathologic process in early-stage IMN and could serve as potential markers for evaluating the disease severity.

## 1. Introduction

Membranous nephropathy (MN) is a common pathological type of nephrotic syndrome in adults. Approximately 30–40% of patients with MN experience gradual progression to chronic kidney disease [1]. MN can generally be divided into idiopathic MN (IMN) and secondary MN according to the different pathogenic factors. IMN accounts for about 75 percent of cases of MN in adults, caused by the deposition of immune complexes of autoantibodies and M-type phospholipase A2 receptor (PLA2R) on the glomerular basement membrane [2, 3].

Macrophage infiltration is a common feature in the inflammatory process initiated by autoantibodies and complement activation. Macrophages play an important role in renal injury [4–6]. Macrophages in the tissue are differentiated from monocytes in the serum. Human peripheral blood monocytes can be classically activated as M1 monocytes and alternatively activated as M2 monocytes [7–10], initially put forward for macrophages. Monocytes highly express CD14, a kind of Toll-like receptor, which can identify monocyte populations. CD163 is a classical symbol of M2 cells. Whereas there is no specific surface marker to identify M1 cells, CD14^+^CD163^−^ cells are considered to be M1-like cells [11]. Another characteristic of activated M2 monocytes is increased expression of CD206 and CD115 [12, 13]. Upon activation, M1 monocytes release proinflammatory cytokines, inducing a Th1 immune response, while M2 monocytes secrete anti-inflammatory mediators, such as interleukin- (IL-) 10, to trigger a Th2 immune response. M1 cells are considered to be antimicrobial and cytotoxic, while M2 monocytes are responsible for tissue repair and have profibrotic action [13, 14].

Previous reports have found that M2 macrophages participated in the pathogenesis of several renal diseases, including anti-neutrophil cytoplasmic antibody- (ANCA-) associated glomerulonephritis, IgA nephropathy [15, 16], proliferative glomerulonephritis [17], and human acute tubulointerstitial nephritis [18], which are closely associated with disease activity in patients with systemic lupus erythematosus (SLE) [19, 20]. Macrophage infiltration is part of the pathological process in IMN. However, the differences in the various types of polarized monocytes/macrophages in IMN have not been clarified.

In this study, we analyzed the counts of different monocyte subpopulations in peripheral blood in patients with early IMN and in healthy controls (HCs). Furthermore, we investigated the potential relationship between different monocyte subsets and the overall laboratory data.

## 2. Materials and Methods

### 2.1. Patients and Controls

A total of 27 patients with newly diagnosed IMN and 16 HCs were recruited for the study at the inpatient service of the Department of Nephrology, the First Hospital of Jilin University (Changchun, China), from January 2017 to December 2017. Patients met the criteria for IMN with pathology-confirmed diagnosis established by the World Health Organization, all in I-II stage histologically. The patients with IMN were classified into three groups according to the 24 h urine protein levels: <4 g, group A (*n* = 9); 4–8 g, group B (*n* = 8); and ≥8 g, group C (*n* = 10). Patients with secondary MN, such as lupus nephritis and other forms of primary nephritis, were excluded from the study. None of the participants had taken immunosuppressive drugs in the previous 6 months, and there was no history of autoimmune and inflammatory diseases, malignant tumors, diabetes mellitus, and atherosclerosis. The 16 age-, sex-, and ethnicity-matched HCs were recruited simultaneously. Written informed consent was provided from all subjects. The Human Ethics Committee of Jilin University approved the study protocol.  Table 1 showed the demographic and clinical characteristics of the participants.

### 2.2. Treatment and Follow-Up

Patients were treated orally with a combination of prednisolone (Tianyao Pharmaceuticals, Tianjin, China) and tacrolimus (Astellas Pharmaceuticals, Ireland). Prednisone was initially administered at 0.5 mg/kg daily for the first two months, and the dose was gradually reduced according to the response. Tacrolimus was taken initially at 0.05 mg/kg daily and adjusted to 5–10 ng/mL in blood. The patients were followed up for 12 weeks. Overall, there were complete records available for six patients, and the other 21 patients were lost during follow-up. Blood samples from the six patients were collected again for subsequent laboratory examinations.

### 2.3. Flow Cytometry Analysis

Venous blood (8 mL) was obtained from all participants. Peripheral blood mononuclear cells (PBMCs) were isolated by density-gradient centrifugation with the Ficoll-Paque PLUS system (Amersham Biosciences, Little Chalfont, UK). PBMCs at a density of 1 × 10^6^/tube were stained in duplicate with the following antibodies: BV510-anti-CD14, PE-anti-CD115 (BD Biosciences, US), PE/Cy7-anti-CD163, and APC/Cy7-anti-206 (BioLegend, US) at 4°C for 30 min. Then, we fixed and permeabilized the cells with a fixation/permeabilization kit (BD Biosciences).

To clarify the function of monocyte subsets, PBMCs (1 × 10^6^/tube) were stimulated in duplicate with lipopolysaccharide, phorbol myristate acetate (50 ng/mL), and ionomycin (1.0 *μ*g/mL, Sigma-Aldrich, St. Louis, US) in RPMI 1640 medium mixed with 10% fetal bovine serum for 2 h at 37°C in 5% CO_2_ and exposed to brefeldin A (GolgiPlug; BD Biosciences) for 4 h, as described in previous studies [9, 10, 21, 22]. Then, the cells were washed, followed by staining with BV510-anti-CD14 and PE/Cy7-anti-CD163. Subsequently, they were fixed and permeabilized and stained with PE-CF594-anti-IL-10 and BV421-anti-IL-12 (BD Biosciences).

Fluorescence minus one (FMO) was used for identifying the positive and negative populations with flow cytometry. The frequencies of different monocyte subsets were assessed on a FACS Calibur instrument (BD, Franklin Lakes, US), and the data were dealt with FlowJo software (v5.7.2; TreeStar; Ashland, US).

### 2.4. Enzyme-Linked Immunosorbent Assay (ELISA)

The serum IL-10 and IL-12 concentrations were measured using ELISA performed with a human IL-10 ELISA kit and a human IL-12 ELISA kit, according to the instructions from the manufacturer (MultiSciences, Hangzhou, China). Briefly, individual serum sample was subjected to ELISA, and the serum IL-10 and IL-12 concentrations were analyzed according to the standard curve established.

### 2.5. Statistical Analysis

Variables were shown as median and range values. We employed the Mann–Whitney *U* nonparametric test to evaluate the differences among groups. The relationship between variables was analyzed by the Spearman rank correlation test. All of the data were carried out with the SPSS version 19.0 software. *P* value of <0.05 represented statistically significant.

## 3. Results

### 3.1. Patient Characteristics

The demographic characteristics did not significantly differ with respect to the distribution of age, sex, serum uric acid concentrations, triglyceride concentrations, total cholesterol concentrations, estimated glomerular filtration rate (eGFR), and monocyte counts among the different groups (Table 1). The levels of 24 h urinary protein and serum PLA2R were significantly higher in early-stage IMN patients than in the HCs, but serum albumin concentrations were lower in the patients with early IMN than in the HCs (*P* < 0.05).

### 3.2. Increased CD14^+^CD163^+^ M2 Monocyte Counts in Patients with Early-Stage IMN

**Figure 1:**
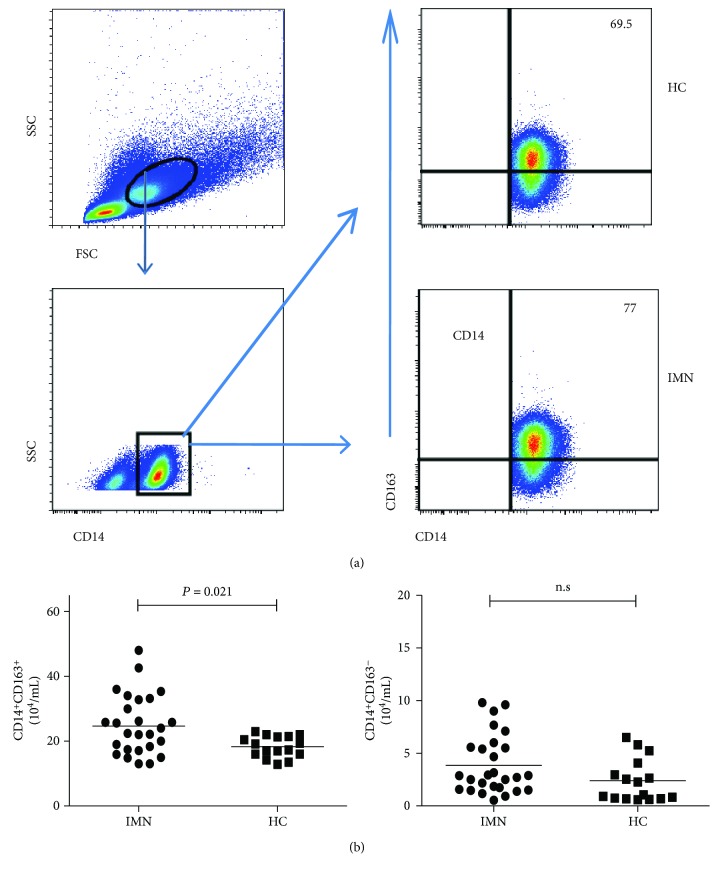
Flow cytometry analysis of the counts of different subpopulations of monocytes. PBMCs from IMN patients and HC were stained with anti-CD14 and anti-CD163. The cells were gated firstly on mononuclear cells and secondly on CD14^+^ monocytes. Afterwards, the counts of CD14^+^CD163^−^ M1-like and CD14^+^CD163^+^ M2-like monocytes were determined by flow cytometry. (a) Representative frequency of monocyte subpopulations. (b) Quantitative analysis of monocyte subpopulations. Horizontal lines show the median values.

We analyzed the circulating CD14^+^CD163^−^ M1-like and CD14^+^CD163^+^ M2-like monocyte counts by flow cytometry. The CD14^+^CD163^+^ M2 monocyte counts were significantly higher in the patients with early-stage IMN than in the HCs (*P* = 0.021; Figure 1), but there were no statistically significant difference among the three IMN subgroups. In addition, the CD14^+^CD163^−^ M1-like monocyte counts did not significantly differ between the IMN patients and controls, or among the three IMN subgroups. The data indicated that patients with incipient IMN had increased CD14^+^CD163^+^ M2-like monocyte counts.

### 3.3. Increased CD14^+^CD163^+^CD206^+^ M2 Monocyte Counts in Patients with Early-Stage IMN

**Figure 2:**
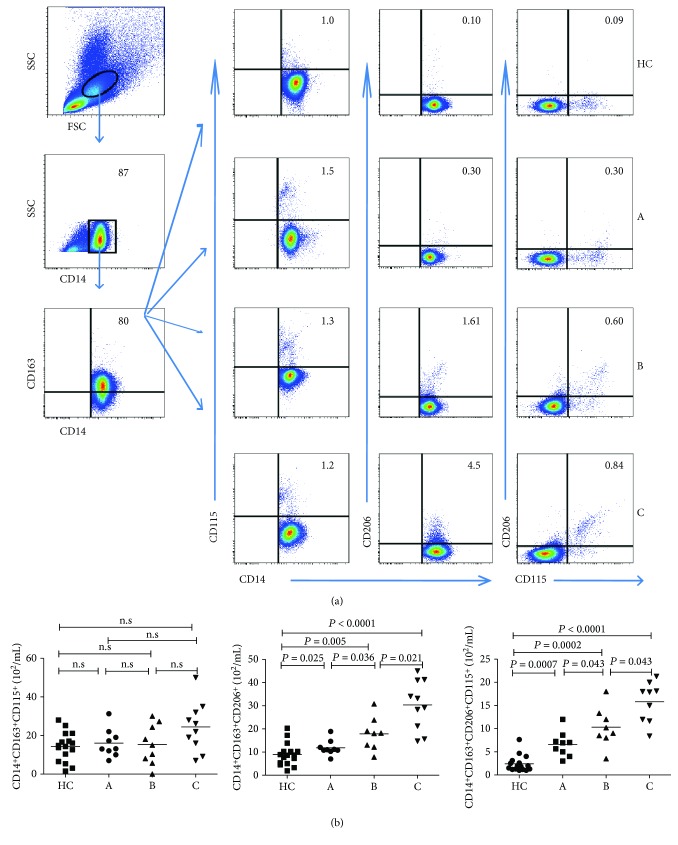
Flow cytometry analysis of CD206^+^ and CD115^+^ M2 monocytes. PBMCs from individual subjects were stained with anti-CD14, anti-CD163, anti-CD206, and anti-CD115. The numbers of CD14^+^CD163^+^CD206^+^, CD14^+^CD163^+^CD115^+^, and CD14^+^CD163^+^CD206^+^CD115^+^ M2-like monocytes were assessed by flow cytometry (a, b). Representative FACS charts and the median values for each group are shown.

Subpopulations of monocytes have distinct surface markers, such as CD206 and CD115, and functions. To figure out the importance of these subpopulations of M2 monocytes, we further analyzed the CD206^+^ and CD115^+^ M2 monocyte counts in patients with IMN and HCs by flow cytometry. The CD14^+^CD163^+^CD206^+^ and CD14^+^ CD163^+^ CD115^+^ CD206^+^ M2 monocyte counts were higher in the patients of early-stage IMN than those in the HCs (Figure 2). However, no significant differences were shown in the CD14^+^CD163^+^CD115^+^ M2 monocyte counts between the patients and the HCs, or among the three subgroups (all *P* > 0.05, Figure 2(b)). Furthermore, the blood CD14^+^CD163^+^CD206^+^ and CD14^+^CD163^+^CD115^+^CD206^+^ M2 monocyte counts were significantly higher in group C patients with IMN than in group B individuals with IMN. Likewise, the counts of those subsets of M2 cells were significantly higher in group B individuals with IMN than in group A subjects with IMN (all *P* < 0.05, Figures 2). Collectively, the increased counts of CD206^+^ M2-like monocytes were positively correlated with the development of IMN.

### 3.4. Intracellular and Serum Cytokine Concentrations in Patients with Early-Stage IMN

**Figure 3:**
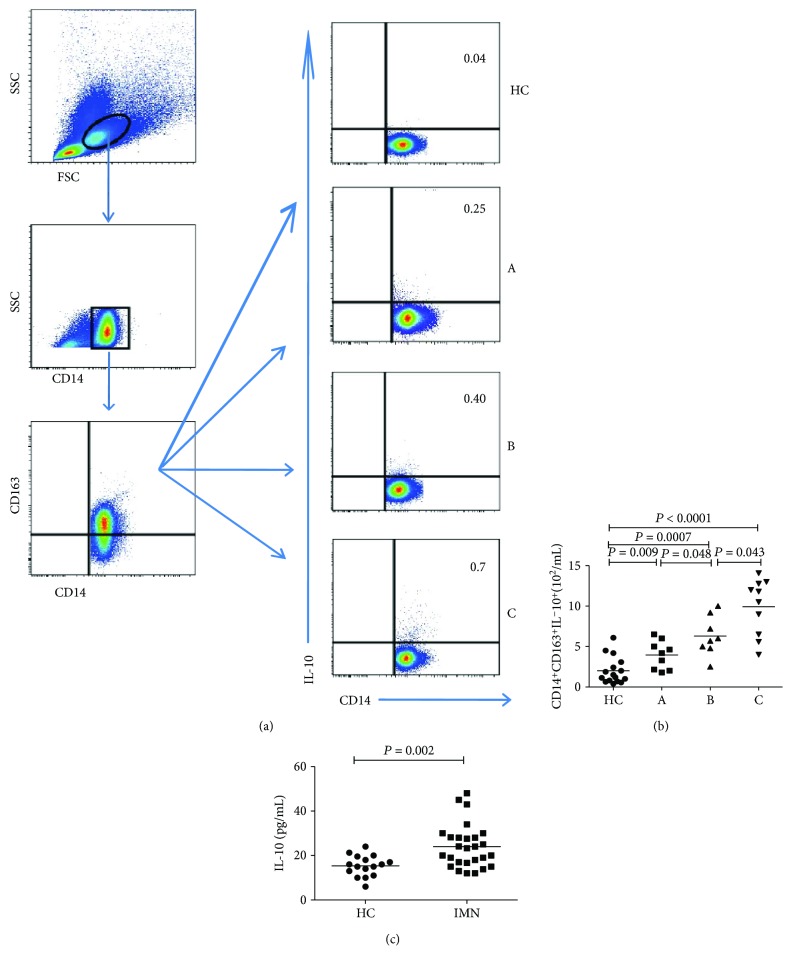
Analysis of IL-10^+^ M2 monocytes and the concentrations of serum IL-10. PBMCs were obtained from all subjects and stimulated in vitro. The cells were stained in duplicate with anti-CD14 and anti-CD163, then fixed and permeabilized, following the staining with anti-IL-10. The numbers of CD14^+^CD163^+^IL-10^+^ M2-like monocytes in all participants were analyzed by flow cytometry. The concentrations of serum IL-10 were determined by ELISA. Data are representative charts or the mean numbers of the monocyte subsets and the mean values of serum IL-10 in all groups. The horizontal lines indicate the median values.

To regulate the function of T cell, M1 monocytes mainly exert a proinflammatory effect by secreting IL-12, whereas M2 monocytes produce IL-10, thereby exerting an anti-inflammatory effect. Therefore, we analyzed the IL-12^+^ M1 and IL-10^+^ M2 monocyte counts, as well as the serum IL-10 and IL-12 concentrations by flow cytometry and ELISA, respectively. As shown in Figures 3(a) and 3(b), patients with IMN had higher IL-10^+^ M2 monocyte counts than the HCs, and the counts were increased in parallel with the severity of IMN (group C > group B > group A; all *P* < 0.05, Figure 3(b)). In contrast, the IL-12^+^ M1 monocyte counts did not significantly differ between patients with IMN and HCs (*P* > 0.05, data not shown). Moreover, the serum IL-10 concentrations were generally higher in patients with IMN than in HCs (all *P* < 0.05, Figure 3(c)), but with no significant differences among the three IMN subgroups. By contrast, the IL-12 concentration did not significantly differ between the patients with IMN and HCs (*P* > 0.05, data not shown).

### 3.5. Correlation between Clinical Parameters and Different Monocyte Subsets

**Figure 4:**
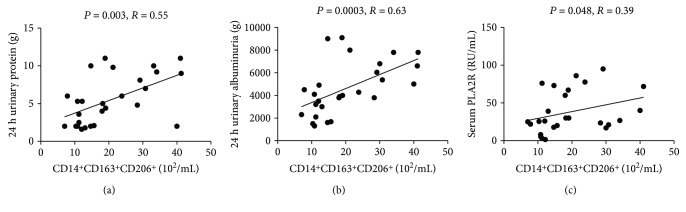
Correlation analysis. The potential correlations between the numbers of monocyte subsets and the values of clinical features in IMN patients were analyzed. The numbers of circulating CD14^+^CD163^+^CD206^+^ M2 cells were positively correlated with the values of 24 h urinary protein (a), 24 h urinary albumin (b), and serum PLA2R (c), respectively. Each point represents an individual subject.

To further understand the roles of monocyte subsets in incipient IMN, we analyzed the potential association between the counts of various monocyte subpopulations and the clinical parameters. CD14^+^CD163^+^CD206^+^ cell counts were positively related with the 24 h urine protein level, 24 h urine albumin concentrations, and serum PLA2R levels (all *P* < 0.05, Figures 4(a)–4(c)). These data revealed that the increased CD14^+^CD163^+^CD206^+^ M2-like monocyte counts were closely associated with the severity of IMN.

### 3.6. Clinical Parameters and Numbers of M2 Monocytes in IMN Patients following Treatment

**Figure 5:**
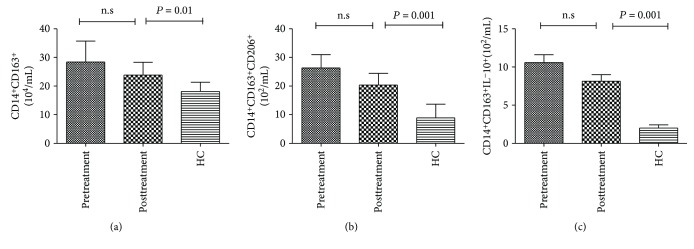
Changes in the counts of M2 monocyte subsets after drug treatment. The counts of different subsets of M2 cells were compared in patients before and after treatment and HCs.

Next, we evaluated clinical responses and quantitative changes of M2 subsets in IMN patients following the prescribed therapy for 12 weeks. As shown in Table 2, the level of 24 h urine protein decreased, while the serum albumin levels increased significantly in these six patients. There was no significant change detected in the values of other clinical indexes. Unfortunately, there were no significant differences in the numbers of circulating CD14^+^CD163^+^, CD14^+^CD163^+^CD206^+^, and CD163^+^CD206^+^IL-10^+^ M2-like monocytes before and after treatment in patients with IMN. However, the counts of these M2 subsets significantly increased after treatment in the patients compared to those in the HCs (all *P* < 0.05, Figure 5).

## 4. Discussion

Monocytes, as progenitors of dendritic cells or macrophages in tissues, are important regulators of the immune response. Similar to macrophages, monocytes can express surface markers with different functions at different stages of renal diseases [13, 23]. Previous observations have indicated that alternatively activated M2 cells are involved in the pathogenic mechanism of some renal diseases by inhibiting inflammatory activity and promoting fibrosis [13, 24, 25]. However, only a few studies have been performed on the association of monocyte phenotypes in the development of IMN.

The current study investigated the numbers of circulating monocyte subpopulations and their potential association with disease severity in patients with early-stage IMN. In the study, the patients had higher CD14^+^CD163^+^ M2 monocyte counts than the HCs. Furthermore, the numbers of CD14^+^CD163^+^CD206^+^ M2 monocyte not only increased in the three subgroups of IMN but also shared the same changes in trend with disease progression. In other words, the CD206^+^ M2 cells were positively correlated with the degree of proteinuria. Similarly, a previous study has shown that CD206^+^ monocytes are associated with proteinuria in acute tubulointerstitial nephritis [18]. Therefore, our findings suggested that the increased CD14^+^CD163^+^CD206^+^ M2-like monocytes may contribute to the pathogenesis of incipient IMN in adults and could serve as a sensitive indicator for evaluating IMN severity. The mechanism may be as follows: macrophage infiltration in tubulointerstitial lesion is a common case in the pathological process in incipient IMN. And the extent of proteinuria is a major determinant of tubulointerstitial damage. We hypothesized that such damage could trigger peripheral CD14^+^CD163^+^CD206^+^ M2-like monocytes, which may be stimulated by IL-4 and IL-13 from Th2-type immune responses in IMN [26, 27], into renal tissues and contribute to tissue repair. Although having protective effect on renal injury, M2 monocytes can secrete fibronectin involved in renal fibrosis [13]. IMN can develop into chronic kidney disease characterized by progressive renal fibrosis. It is possible that increased CD14^+^CD163^+^CD206^+^ monocytes have profibrotic effect while they ameliorate proteinuria in IMN. However, the hypothesis should be investigated in detail in future studies.

IL-10 is an important cytokine with respect to its effect on decreasing inflammation and triggering immunosuppressive activities [28]. Our observations have revealed upregulated concentrations of serum IL-10 in early-stage IMN patients, consistent with the previous reports [29, 30]. Furthermore, intracellular IL-10 concentrations in CD14^+^CD163^+^ M2 monocytes were found to be positively correlated with protein progression, but the serum IL-10 concentrations did not seem to be significantly associated with it. The positive relationship may reflect a functional response corresponding to increased numbers of CD14^+^CD163^+^CD206^+^ M2-like monocytes. This result likely reflects the fact that serum IL-10 is also released by other immunocytes, such as Th2, regulatory T cells, and T follicular helper cells [29, 30], besides M2 monocytes. Above all, IL-10^+^ M2 cells might be another useful indicator for evaluating the severity of incipient IMN.

The PLA2R levels have been clinically used as good biomarkers for evaluating IMN development, progression, and recurrence. Therefore, the association between monocyte subsets and clinical features in patients with incipient IMN was analyzed. The CD14^+^CD163^+^CD206^+^ M2-like monocyte counts in the patients with IMN were positively correlated with the 24 h urine albumin and serum PLA2R levels, further supporting that the CD14^+^CD163^+^CD206^+^ M2-like monocytes may play a potential role in the pathogenesis of IMN in early stage. And these subsets could provide sensitive markers for disease severity of IMN.

After 12 weeks of follow-up, the therapy resulted in partial remission in six patients with IMN based on the levels of 24 h urine protein. Although there were no differences in the numbers of M2 subsets before and after treatment, circulating CD14^+^CD163^+^CD206^+^ M2-like cells in the patients after treatment were significantly greater than those in HCs, indicating that CD14^+^CD163^+^CD206^+^ M2-like cells may be involved in the pathogenic process of IMN. The lack of statistical significance may stem from the small group of patients and/or the short follow-up period. Therefore, we will further study these effects with more patients and over a long-term follow-up period beyond 6 months. We also recognized other limitations of this preliminary study, such as the lack of functional analysis of CD206^+^ M2-like cells. Accordingly, we are planning to conduct more detailed analyses of the role of M2 monocytes using a cell culture model and murine model of IMN.

In conclusion, the present findings demonstrate that the immune status of patients with early-stage IMN is closely associated with CD14^+^CD163^+^CD206^+^ M2-like monocytes. Thus, CD14^+^CD163^+^CD206^+^ M2-like monocytes may serve as sensitive indicators for evaluating the severity of IMN. Nevertheless, the mechanism underlying the effect of CD206^+^ M2 monocytes in the pathogenesis of IMN requires further study.

## Figures and Tables

**Table 1:** The demographic and clinical characteristics of participants.

	IMN group A *n* = 9	IMN group B *n* = 8	IMN group C *n* = 10	HC *n* = 16
Age, years	38 (27–58)	45 (28–64)	49 (25–60)	42 (25–63)
Female/male	5/4	3/5	6/4	7/9
Monocytes (10^9^/L)	0.3 (0.2–0.45)	0.32 (0.21–0.5)	0.33 (0.2–0.6)	0.35 (0.18–0.35)
Serum uric acid (*μ*mol/L)	311 (163–450)	363 (300–424)	376 (235–499)	330 (230–410)
Triglycerides (mmol/L)	1.6 (0.6–3.77)	2.0 (0.87–5.34)	2.1 (1.66–5.26)	1.3 (0.42–1.78)
Total cholesterol (mmol/L)	6.8 (5.8–13)	6.9 (4.4–9)	7.4 (4.8–9.1)	3.9(2.8–5.6)
Serum albumin (g/L)	25 (19.4–39)^∗^	22 (15–30)^∗^	21 (17–27)^∗^	42 (40–52)
Urinary proteins (g/24 h)	2 (2–5.3)^∗^	5.65 (4.4–7) ^∗^	9.8 (8–11)^∗^	0.01 (0–0.05)
eGFR (CKD-EPI) (mL/min/1.73 m^2^)	119 (99–127)	108 (96–124)	99.5 (90–125)	118 (102–130)
Serum PLA2R (RU/mL)	17.8 (3–76)^∗^	26 (1.6–77.7)^∗^	72 (16.9–170)^∗^	1.5 (0–2.7)

Data shown are median and range. Normal values: monocytes: 0.1–0.6 (10^9^/L), serum uric acid: 155–357 (*μ*mol/L), triglycerides: 0.28–1.8 (mmol/L), total cholesterol (mmol/L): 2.6–6.0 (mmol/L), serum albumin: 40.00–55.00 (g/L), urinary proteins: 0–0.2 (g/24 h), eGFR: 80–120 (mL/min/1.73 m^2^), and serum PLA2R: 0–14 (RU/mL). IMN: idiopathic membranous nephropathy; HC: healthy control; eGFR: estimated glomerular filtration rate. ^∗^*P* < 0.05 versus the controls.

**Table 2:** Effect of treatment on the values of clinical measures in follow-up IMN patients.

	Before treatment	After treatment
Age, years	39 (28–58) 3/3	
Female/male		
Monocytes (10^9^/L)	0.3 (0.2–0.4)	0.6 (0.4–0.8)^∗^
Serum uric acid (*μ*mol/L)	300 (166–450)	320 (180–420)
Triglycerides (mmol/L)	2.5 (0.7–3.6)	1.9 (0.97–4)
Total cholesterol (mmol/L)	6.5 (5.8–10)	6 (4.3–7.8)
Serum albumin (g/L)	25 (18–28)	30 (26–35)^∗^
Urinary proteins (g/24 h)	7.8 (2.5–10)	1.6 (0.5–3)^∗^
eGFR (CKD-EPI) (mL/min/1.73 m^2^)	110 (95–120)	118 (106–125)

Data are presented as median range. ^∗^*P* < 0.05 versus the values before treatment.

## Data Availability

The data used to support the findings of this study have not been made available due to patient privacy.
